# Supplementary material for: A high-throughput RNAi screen for detection of immune-checkpoint molecules that mediate tumor resistance to cytotoxic T lymphocytes
Source: EMBO Mol Med. 2015 Feb 17;7(4):450–63. doi: 10.15252/emmm.201404414 (PMC4403046; doi:10.15252/emmm.201404414)
Supplement: Supplementary file 1 — Supplementary Information [file emmm0007-0450-sd1.pdf]

Supplementary Data

Table of Contents:

- Supplementary Methods
- Supplementary Figures S1(A-B)
- Supplementary Figures S2 (A-B)
- Supplementary Figures S3
- Supplementary Figures S4(A-B)
- Supplementary Figures S5
- Supplementary Figures S6(A-B)
- Supplementary Figures S7(A-B)

Supplementary Methods

Immunoblot and RT-PCR:

Whole cell protein extracts were prepared for western blot analysis and the immunoblots were probed with the following antibodies: anti-human CCR9 antibody (MAB179; R&D systems), anti-human PD-L1 (MAB156, R&D systems), anti-human beta-actin (Abcam), anti-phospho-STAT1 (Tyr701) (Cell Signalling Technology). For RT-PCR, total RNA was extracted from cell pellets using the RNeasy Micro kit (Qiagen, Hilden, Germany) and 1 μg RNA from each sample was reverse transcribed using the QuantiTect reverse transcription kit (Qiagen) as instructed by the manufacturer. Primer sequences for PCR run: CCR9 Forward: 5'- CAGTGAACCCCTGGACAACT-3', CCR9 Reverse: 5'-TGCCACTCAACAGAACAAGC-3', PD-L1 Forward: 5'- GTACCTTGGCTTTGCCACAT-3', PD-L1 Reverse: 5'-CCAACACCACAAGGAGGAGT-3', GAPDH Forward: 5'-GAGTCAACGGATTTGGTCGT-3', GAPDH Reverse: 5'-TTGATTTTGGAGGGATCTCG-3'.

Chromium-release cytotoxicity assay:

For the chromium (Cr) -release cytotoxicity assay, tumor cells were transfected with described siRNAs (obtained from Dharmacon, GE Healthcare as SMARTpool or individual siRNA sequences) or plasmids as described in Methods before. 72 hours later, the cells were harvested, washed and labeled with 200μL $^{51}Cr/10^{6}$ target cells (Perkins-Elmer, Germany) for 45 min at 37°C. After labeling, the cells were carefully washed thrice to remove cell-free chromium and 3000 target cells /well were cocultured with T cells in 96 well plates at a T cell to target cell (E:T) ratio of 1:1 to 100:1 for 4 hours at 37°C. After 4 hours, the plates were spun down and the supernatant was harvested for measuring the radioactivity released by dead cells using the Gamma counter (Cobra counter Packard, Perkin Elmer, Rodgau, Germany). As a control for spontaneous release, the labeled cells were co-incubated with

media alone; for maximum release, the cells were incubated with 10% Triton X-100 instead of T cells. % specific lysis was then calculated by the formula given below:

% specific lysis = (Experimental release - Spontaneous release)/ (Maximum release - Spontaneous release) x 100

Imprinting T cells with immunosuppressive tumor supernatants:

To assess whether CCR9 mediates suppression on T cells via soluble mediators, MCF7 tumor cells were reverse transfected with control or CCR9-specific siRNA as described before. After 60 h, cell culture supernatants (SSN) were harvested from both the knockdown conditions and used to culture $1x10^7$ fresh survivin-specific T cells, each with the respective supernatants overnight. On the following day, knocked down MCF7 tumor cells ($CCR9^{hi}$ and $CCR9^{lo}$) were harvested and used as target cells, along with wild type MCF7 cells, and the respective supernatant treated T cells ($CCR9^{hi}$ and $CCR9^{lo}$ SSN treated TCs) were used as effector cells in the classical Cr-release assay.

ELISpot assay:

Briefly, CCR9 expression was inhibited in the tumor cell lines using specific siRNAs, along with control knockdown. 48 hours post siRNA transfection, the knocked down cells were harvested, washed and cocultured with either survivin-specific T cells (5000 cells) or polyclonal CD8+ T cells (10,000 cells) and anti-EpCAM x CD3 antibody in ELISpot wells at a ratio of 5:1 effector to target cells for 24 hours. IFN-γ or granzyme B spots were developed using the Mabtech (Sweden) kit and measured using the enzyme-linked immunospot (ELISPOT) software (CTL Europe, Bonn, Germany). Statistical comparison was performed from three test wells per group.

ELISA:

To measure CCL25 levels in different tumor cell lines, sandwich ELISA was performed with 25 µg of protein lysates from the respective tumor cell lines using the commercial CCL25 ELISA kit (R&D systems). CCL25 protein standards were used as positive control and rhPD-L1 protein (BioLegend) was used as a negative control to check for unspecific binding of

anti-CCL25 antibody coated plates. Experiments were performed in triplicate wells for statistical comparison.

Cytokine measurements:

MCF7 cells were transfected with CCR9-specifc or control siRNA for 48 hours and then harvested and cocultured with 10^4 survivin-specific T cells at 1:5 ratio in 96-well plate for additional 24 hours at 37°C. Three test wells per group were included in this study. After incubation, the plates were spun down and 100 µl of the culture supernatant was collected from each test well and centrifuged at 1000xg for 15 min at 4°C. The clear supernatant was collected and used directly for cytokine measurement using the Bio-Plex Pro Assay kit as described by the manufacturer (Biorad, Germany). Data was analyzed using the Bio-Plex Manager software version 6.0.

Phospho-plex analysis:

MCF7 cells were transfected with either control or CCR9-specific siRNAs as described above. After 72 hours, the cells were harvested and $8x10^4$ cells in 100 µl of cytokine free X-vivo 20 medium were plated per well of a 96-well plate. To this $2x10^6$ survivin-specific T cells, suspended in 100µl of X-vivo medium, were added. The tumor and T cells were cocultured for 1 min, 5 min and 20 min for T cell receptor complex analysis and for 20 min, 1 hour and 2 hours for phospho-STAT analysis. As positive control for T cell activation for phopho-TCR analysis, TCs were stimulated with PMA (50 ng/ml) and ionomycin (500 ng/ml). After respective co-incubation time points, each cell group was added to 100µl of pan mouse IgG beads (Invitrogen, Germany) coated with 4µg of anti-EpCAM antibody (clone HEA125, provided by G. Moldenhauer, DKFZ, Germany) for 30 minutes at 4°C to separate EpCAM+ MCF7 tumor cells from EpCAM- T cells. Bead-separated TCs were then lysed and total protein concentration was measured using BSA Protein Assay Kit (Thermo scientific, Rockford, U.S.) and this was normalized across all samples before phosphoprotein detection using the 7-plex T cell receptor signaling phosphoprotein kit or phospho-STAT 5-plex kit

(Millipore, Billerica, U.S.), as instructed by the manufacturer. Measurements were performed using Luminex100 Bio-Plex System (Luminex, Austin, U.S.) and all the data were analysed using Bio-Rad Bio-Plex Manager software version 4.1.1 (BioRad Life Science Research, Hercules, U.S.).

Transwell migration assay:

Migration assay was performed in 24-well plates with 8 µm transwell inserts. The receiver well was cultivated with 500 µl of medium alone or medium supplemented with 3 µg/ml of rhCCL25 protein (R&D systems). Transwell inserts (Greiner bio-one) containing $1x10^5$ MDA-MB-231 cells in 200 µl DMEM medium, supplemented with or without pertussis toxin (2 µg/ml), were overlaid onto the receiver wells and incubated for 24 h. Cells that migrated into the lower chamber were carefully harvested after 24 h and quantified using the CellTiter-Glo (CTG) assay (Promega) as detailed in the manufacturer's protocol. Experiment was performed in triplicates per sample group.

Flow cytometry:

For flow cytometric analysis, all samples were acquired with the FACS Canto II Cell Analyzer (BD Biosciences) and data was analyzed using FlowJo software version 8.8 (Tree Star). FITC labeled anti-human HLA-A2 (BB7.2), Alexa-647 labeled anti-human CCR9 and respective isotype control antibodies were obtained from BD Biosciences.

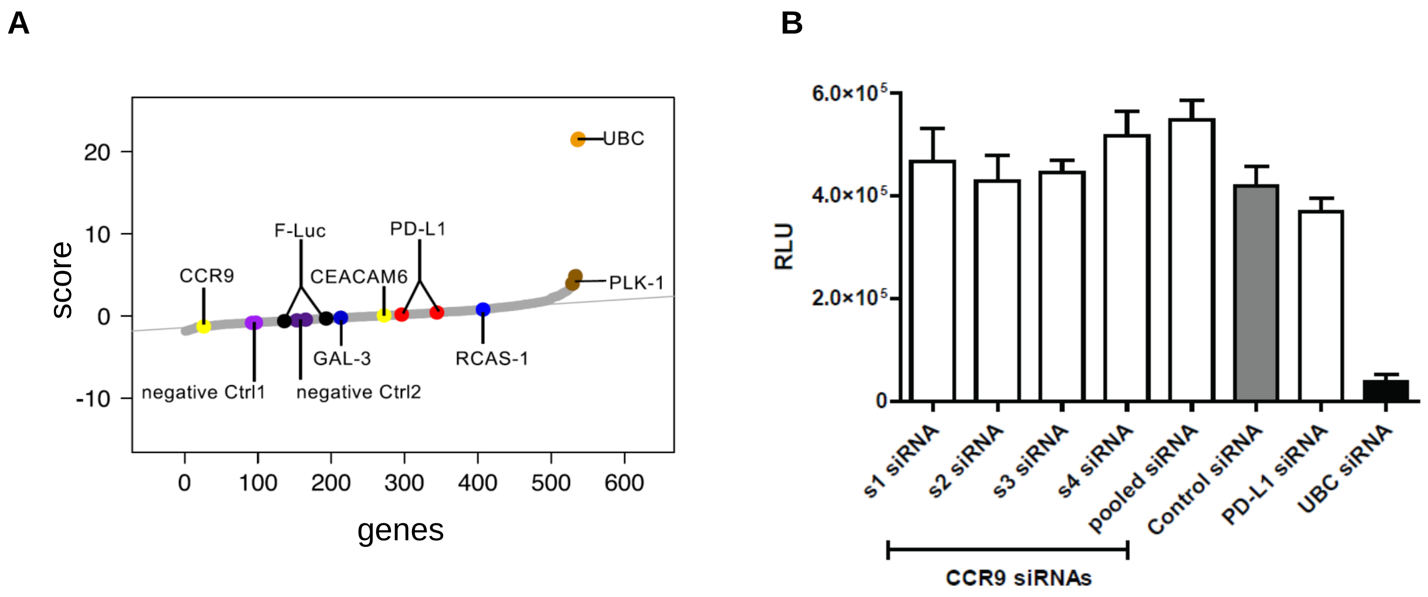

Supplementary Figure S1. Impact of gene knockdowns on the viability of MCF7 cells. (A) Normalized score for all the tested genes depicted for the CellTiter-Glo (CTG) assay that was used to determine lethal genes that directly affect MCF7 cell viability upon knockdown. **(B)** CTG viability assay with MCF7 cells upon CCR9 knockdown using individual (s1-s4) and pooled siRNAs, along with lethality control (UBC), positive immunosuppressive control (PD-L1) and negative non-specific (control) siRNA.

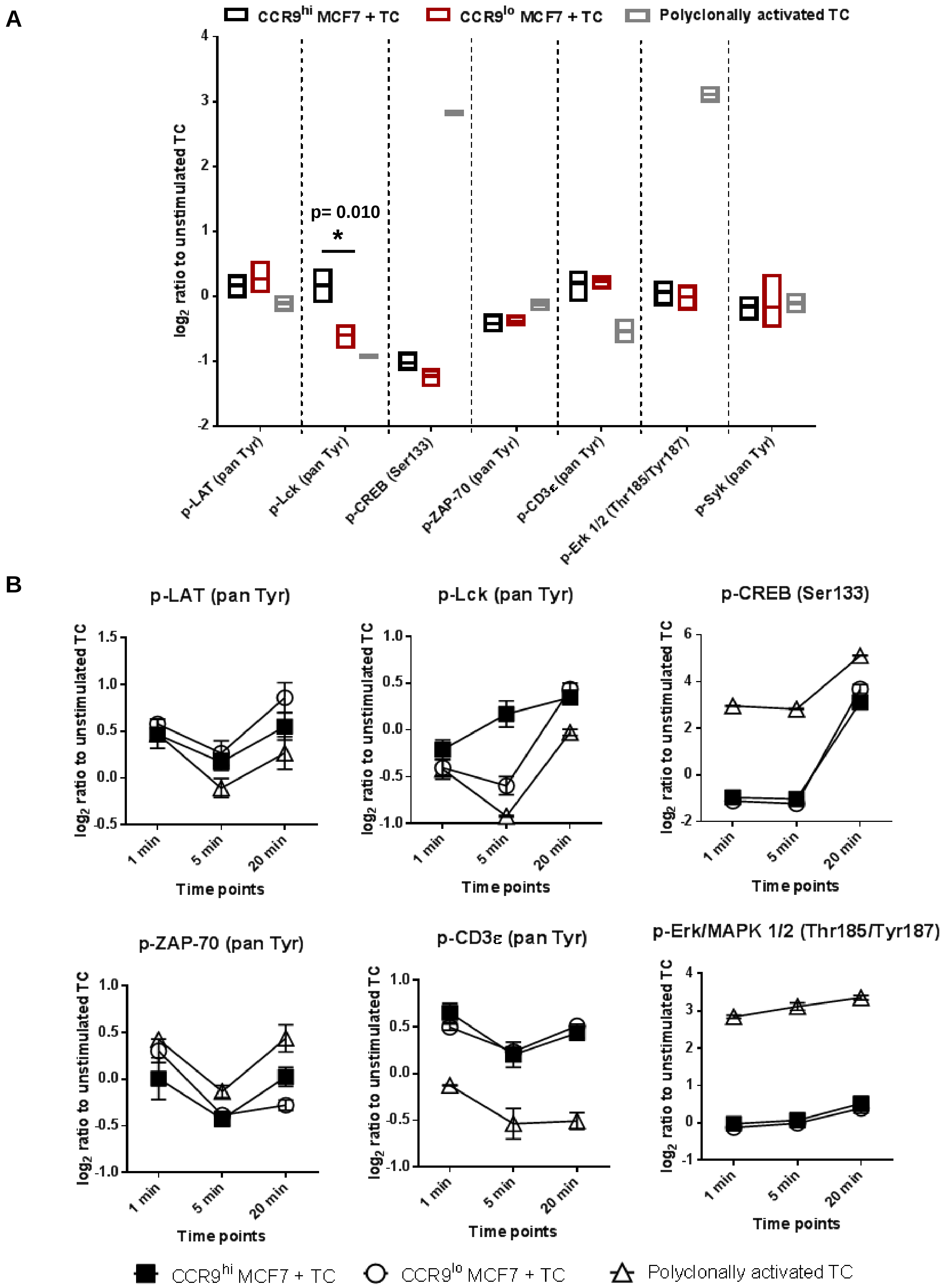

Supplementary Figure S2. Impact of CCR9 on TCR signaling. (A-B) Phosphorylated levels of the activated T cell receptor signaling components were assayed in survivin-specific T cells (TC) that were co-cultured with CCR9^{hi} or CCR9^{lo} MCF7 cells for 1 min, 5 mins or 20 mins. As positive control for activation, survivin TC were polyclonally stimulated with PMA and ionomycin alone. Log$_2$ ratio of mean fluorescent intensity (MFI) of specified phospho-proteins to the unstimulated survivin TCs are depicted for the 5 minute time point in **A** and time-dependent kinetics for individual analytes are shown in **B.** Experiments were performed in triplicates in three independent repeats and error bars denote +/- SEM. Unpaired, two-tailed student *t*-test was used to assess the statistical significance.

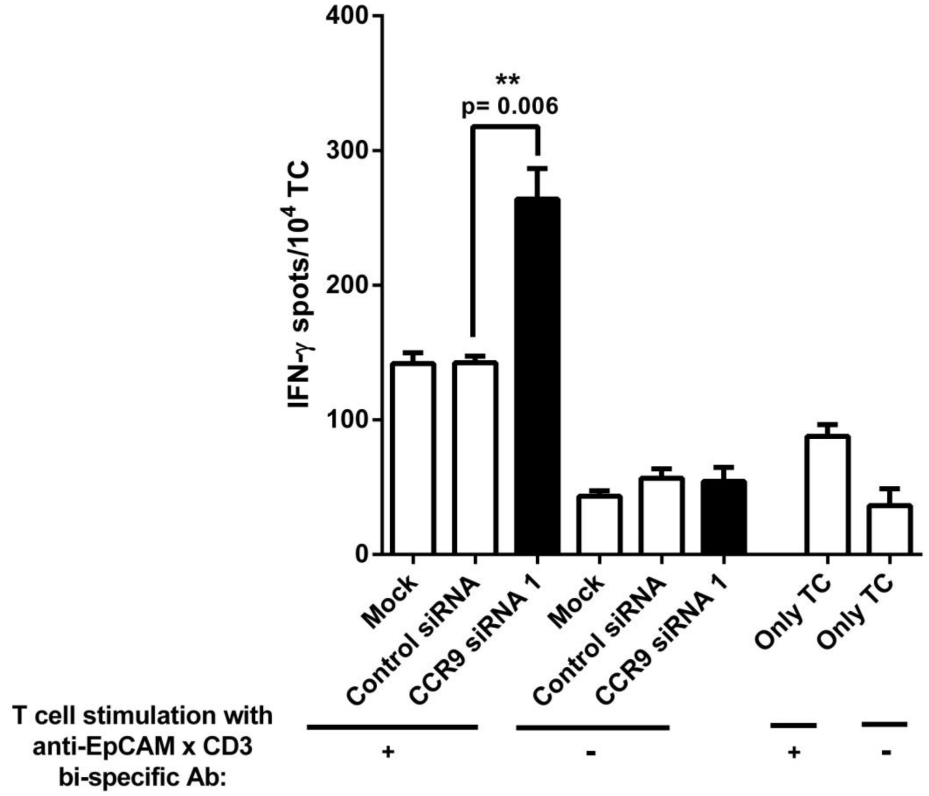

Supplementary Figure S3. CCR9-mediated immune suppression requires TCR engagement. MCF7 tumor cells were transfected with mock, control or CCR9-specific siRNA and subsequently used for IFN-γ ELISpot assay along with PBMC-derived CD8+ T cells in the presence or absence of cross-linking anti-EpCAM x CD3 bi-specific antibody. Mean IFN-γ secretion by CD8+ T cells, as spot numbers, are represented herein; error bars denote +/- SEM. n=3. *P* value was calculated using the unpaired, two-tailed student *t*-test

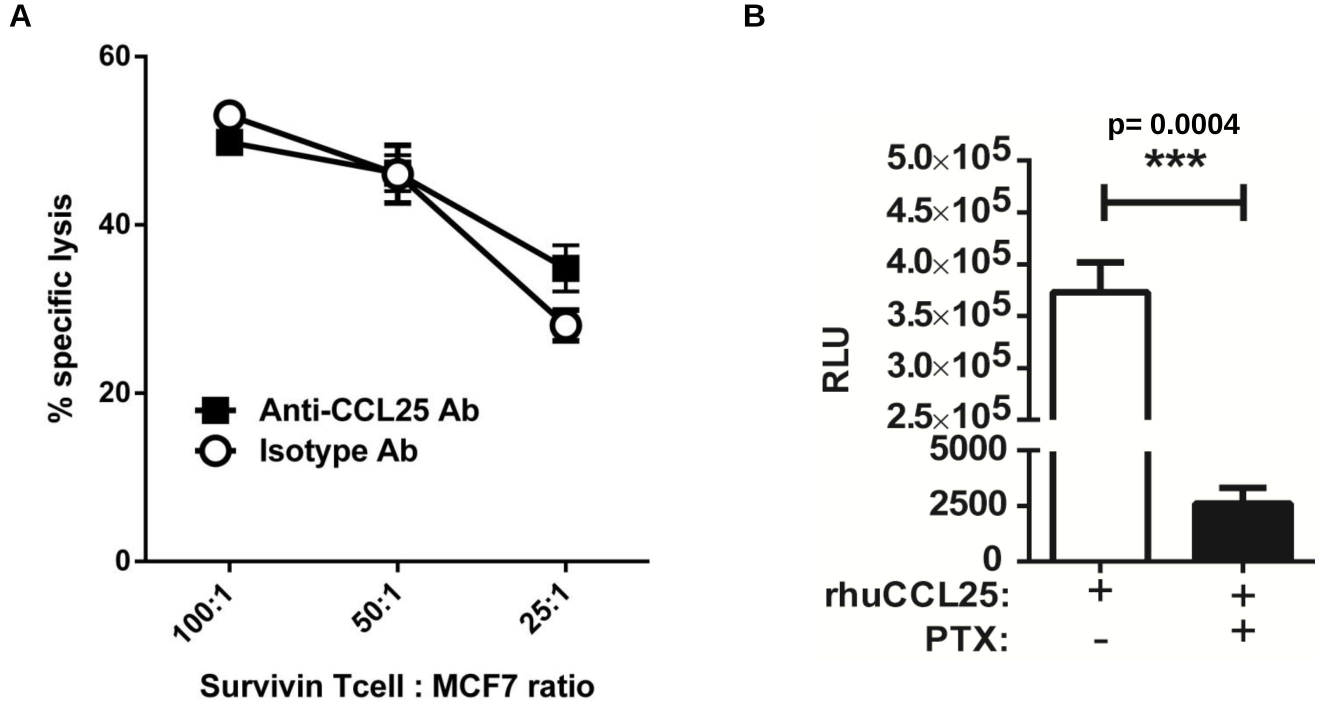

Supplementary Figure S4. Role of CCL25 in immune-suppression of antigen-specific T cells. (A) Cr-release assay showing % specific lysis of MCF7 cells by survivin TC upon CCL25 inhibition using blocking antibody or the control isotype antibody. Curves represent mean ± SEM. **(B)** Transwell migration assay showing migration of MDA-MB-231 tumor cells in response to rhCCL25 protein in the presence or absence of pertussis toxin (PTX). Migrated cells were fixed and stained with CTG dye and luminescence was used to score the migration capacity. Error bars denote SEM; n=3 and statistical significance was calculated using the unpaired, two-tailed student *t*-test.

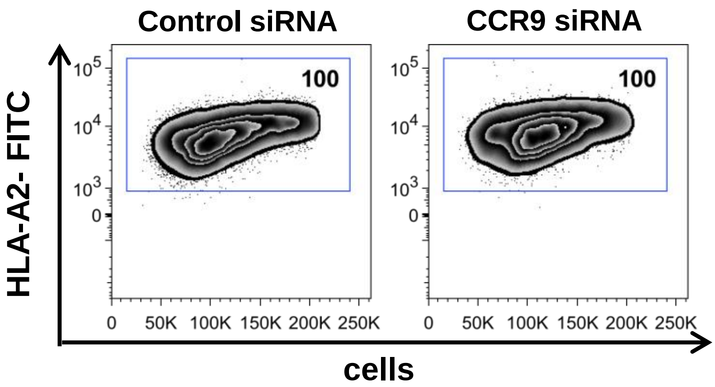

Supplementary Figure S5. CCR9 does not alter MHC-I expression on tumor cells. Flow cytometric analysis showing surface expression of HLA-A2 on MDA-MB-231 tumor cell lines 72 h post-transfection with control or CCR9-specific siRNA. Gates were set based on the isotype control antibody and the numbers represent the proportion (%) of HLA-A2 positive cells amongst the total live cell population.

A

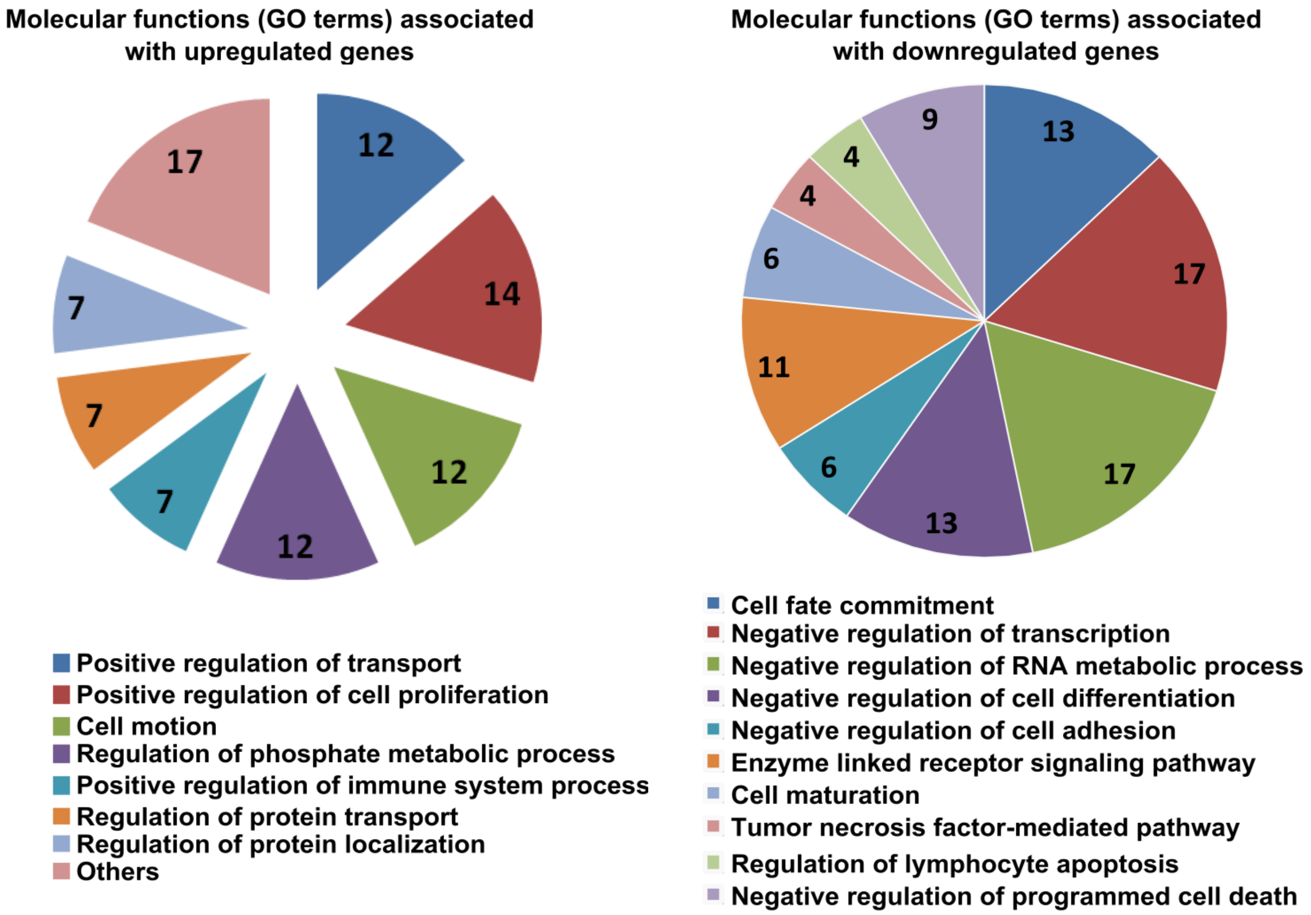

B

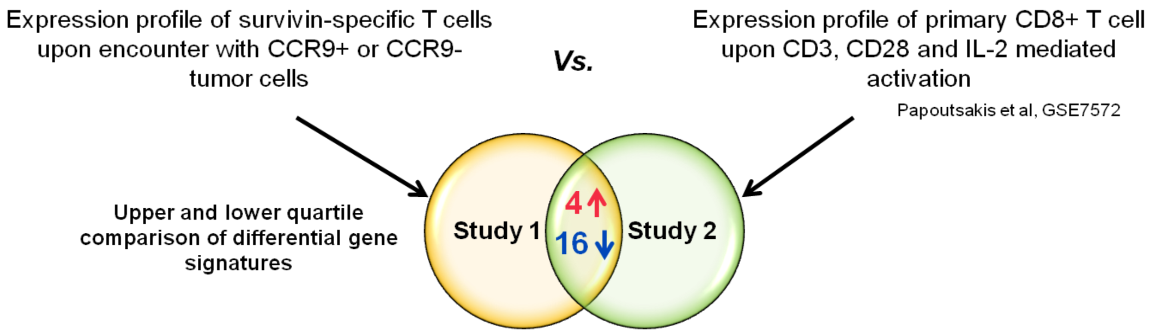

Upregulated	Function
CCND2	Cyclin D2, expressed in response to IL2R triggering
IL2RA	Binds to IL2, triggers cyclin D2 via STAT5, induces T cell proliferation upon activation
CISH	Promotes TCR mediated proliferation and prolongs survival of activated T cells
LTA	Lymphotoxin produced by CTLs, toxic to tumors

Downregulated	Function
MYLIP	E3 ubiquitin-protein ligase; degradation of myosin regulatory light chain
TMEM107	Transmembrane protein 107
TUBB2A	Tubulin, beta 2A class IIa
YPEL5	Yippee-Like 5
TOB1	Inhibits T cell proliferation and downregulates IL-2; loss of TOB1 reduces the threshold for T cell activation
EGR1	Early growth response 1; transcriptional regulator involved in synapse plasticity
ID1	Restricts T cell potential; induces apoptosis in effector cells
PPM1D	Phosphatase; negatively regulates the activity of p38 MAPK

Supplementary Figure S6. CCR9-induced gene expression signature in T cells. (A) Enrichment analysis showing the percentages of differentially expressed genes in CCR9^{lo} treated TCs that are attributed to a certain GO term, with only statistically significant enrichment terms being plotted. **(B)** Overlapping gene signatures between the CCR9-induced expression profile and a published study comparing gene expression changes in CD8 T cells before and after activation (GSE7572; refer methods). Significantly up- and down-regulated genes (in the top 30%) in both the studies were compared and few of the common gene signatures are summarized in brief.

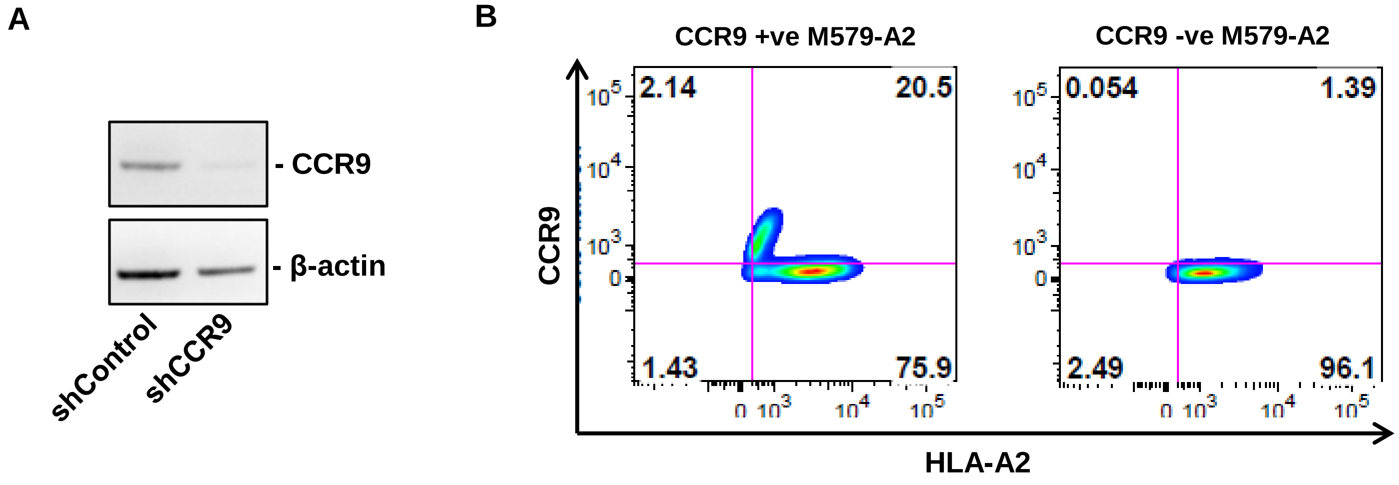

Supplementary Figure S7. Stable knockdown of CCR9 in melanoma M579-A2 cells. (A) Immunoblot showing knockdown of CCR9 in M579-A2 cells transduced with lentiviral particles encoding either control shRNA (shControl) or CCR9-specific shRNA (shCCR9). Anti- β-actin antibody was used for loading control. **(B)** Flow cytomeric staining showing surface expression of CCR9 (y-axis) and HLA-A2 (x-axis) on M579-A2 cells that were transduced with shControl or shCCR9. Gates were set based on the respective isotype control antibodies and the numbers represent the proportion (%) amongst the total live cell population.
