# Supplementary material for: A high-throughput RNAi screen for detection of immune-checkpoint molecules that mediate tumor resistance to cytotoxic T lymphocytes
Source: EMBO Mol Med. 2015 Feb 17;7(4):450–63. doi: 10.15252/emmm.201404414 (PMC4403046; doi:10.15252/emmm.201404414)
Supplement: Supplementary file 3 — Source Data for Figure 5E [file emmm0007-0450-sd3.pdf]

Source Data

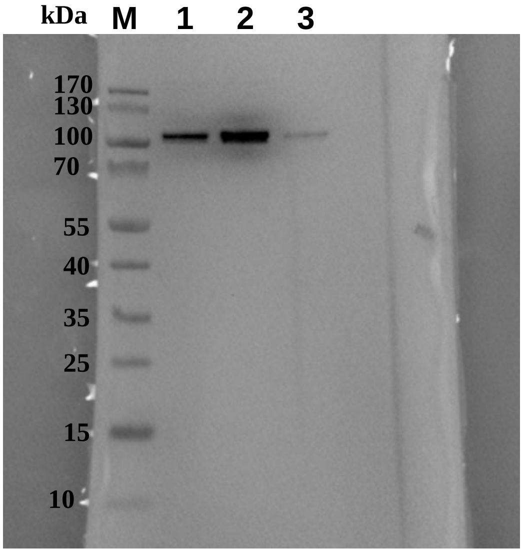

pSTAT1 (Tyr701) Ab

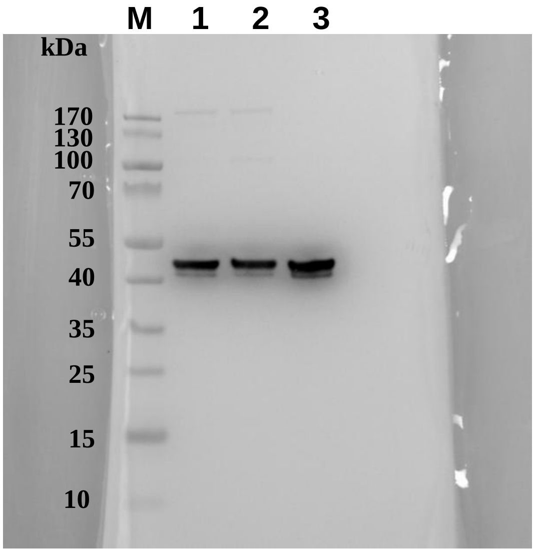

β-actin Ab

Legend:

M: Molecular weight marker

Lane 1: CCR9^{hi} treated TC

Lane 2: CCR9^{lo} treated TC

Lane 3: Unstimulated TC

Figure 5E
